# Rhinovirus Genotypes Circulating in Bulgaria, 2018–2021

**DOI:** 10.3390/v15071608

**Published:** 2023-07-22

**Authors:** Irina Georgieva, Asya Stoyanova, Svetla Angelova, Neli Korsun, Savina Stoitsova, Lubomira Nikolaeva-Glomb

**Affiliations:** 1 Department of Virology, National Centre of Infectious and Parasitic Diseases, 1233 Sofia, Bulgarialubomira@gmail.com (L.N.-G.); 2 Clinic of Infectious Diseases, University Hospital “Prof. Dr. Stoyan Kirkovich” AD, 6003 Stara Zagora, Bulgaria

**Keywords:** rhinovirus, genotype, sequencing

## Abstract

Rhinoviruses (RV) are one of the most common causative agents of respiratory infections, with significant socioeconomic impact. RV infections are not notifiable in Bulgaria, and little is known about the different RV genotypes circulating in the country. This study aims to investigate the diversity of RV genotypes that were circulating in Bulgaria in the period 2018–2021 in samples from ILI/ARI patients. Genotype assignment was based on sequencing and phylogenetic analysis of the 5′ untranslated region and the VP4-VP2 region. Out of a total of 1385 nasopharyngeal swabs tested, 166 were RV-positive (RV detection rate: 11.99% (166/1385)). Those with a cycle threshold <25 were selected for genotyping (n = 63). RV isolates were successfully genotyped and classified into 34 genotypes within *Rhinovirus A* (RV-A)*, Rhinovirus B* (RV-B) and *Rhinovirus C* (RV-C) species. Presumptive recombination events between the 5′UTR and VP4-VP2 regions were detected in three of the isolates. RV-A and RV-C were the prevalent RV species, with significantly more frequent detections of RV-A in the years before the COVID-19 pandemic compared to the post-pandemic period, when RV-C prevailed. The present study is the first to determine RV genotypes in Bulgaria and the circulation of RV-C has been described for the first time in the country.

## 1. Introduction

Rhinoviruses (RVs) are the causative agents for more than half of upper respiratory tract infections [1]. Rhinovirus (RV) infections are considered benign, self-limited and generally mild, but the mere fact that they are so common renders a significant economic impact on the health system and the quality of life [2]. Although the upper respiratory tract is the most common site of the RV infection, RVs were associated with lower respiratory tract complications such as bronchitis, bronchiolitis and pneumonia [3].

RVs are an extremely heterogeneous group of viruses belonging to the *Enterovirus* genus within the *Picornaviridae* family. To date, over 165 RV genotypes have been described. Based on their genetic similarities, RVs are classified into three species designated as *Rhinovirus A* (RV-A)*, Rhinovirus B* (RV-B) and *Rhinovirus C* (RV-C) (International Committee on Taxonomy of Viruses (ICTV): https://ictv.global/taxonomy/, accessed on 4 May 2023). The RV genome is a single positive-stranded RNA of approximately 7.2–7.5 kb in size. The genetic information is coded in a single open reading frame flanked by two untranslated regions (UTRs). The molecular classification of RVs into genotypes is based on the nucleotide sequence of either the 5′UTR, VP1 or the VP4/VP2 genome region [4,5,6].

In Bulgaria, influenza and acute respiratory infections are monitored by a national sentinel surveillance network that involves general practitioners and pediatricians working from 218 outpatient healthcare facilities in all the 28 administrative regions of the country and serves 381,493 people from all age groups (5.3% of the country’s population). The surveillance includes laboratory investigations of cases that meet the diagnostic criteria of influenza-like illness (ILI) and acute respiratory illness (ARI). This monitoring system provides important information about the beginning, duration and the end of the flu season, as well as the types of influenza viruses circulating in the country. In addition, hospitals and primary care settings send, at their discretion, samples from symptomatic patients to the National Reference Laboratory (NRL) for Influenza and ARI at the National Centre of Infectious and Parasitic Diseases for virological testing. RV infection is not notifiable in Bulgaria, and the European Centre for Disease Prevention and Control (ECDC) does not routinely collect data on RVs. The surveillance system established in Bulgaria does not provide detailed information about the different RV types circulating in the country.

This study aims to investigate the diversity of RV genotypes in Bulgaria, with consideration given to relative disease severity and clinical diagnoses of confirmed RV cases.

## 2. Materials and Methods

### 2.1. Study Population and Specimen Collection

A total of 1385 respiratory samples from symptomatic patients meeting the criteria for ILI or ARI in primary care settings or hospitals and from sentinel surveillance were enrolled in this study. The samples were obtained mainly during the cold months of the year, when there is an increased incidence of respiratory infections. The samples included in this study were collected between 2018 and 2021. In 2020, the majority of samples received at the NRL for Influenza and ARI were tested solely for SARS-CoV-2 and therefore were excluded from the study. Samples obtained in 2021 did not include accompanying information on the diagnosis and were therefore designated in this study as ILI/ARI-possible COVID-19.

Combined nasal and throat specimens from the enrolled patients were collected with the help of commercial polyester collection swabs and virus transport media. The swabs were stored at 4 °C for up to 72 h before shipment to the laboratory. Specimens were processed immediately or stored at −80 °C before testing.

### 2.2. Clinical Data and Statistics

The NRL for Influenza and ARI collects individual information on diagnosis and demographics identified by physicians. RV positivity rate (RV positive over total samples tested) was calculated. Clinical features and the incidence of each RV type, as well as the detection rate of different RV types before and after the COVID-19 pandemic, were compared using the Fisher’s exact test for categorical variables. The *p*-values < 0.05 were considered statistically significant.

### 2.3. Extraction of Nucleic Acids and Detection of RV

Viral nucleic acids were extracted automatically from 400 µL of the respiratory specimens using a commercial ExiPrep Dx Viral DNA/RNA kit (Bioneer, Daedeok-gu, Daejeon, Republic of Korea) in accordance with the manufacturer’s instructions.

Real-time reverse transcription polymerase chain reaction (RT-PCR) was employed for RV detection using specific primers/probes and Applied Biosystems™ AgPath-ID One Step RT-PCR Kit in 25 μL reaction volume. The primers and probe were directed to the 5′UTR as described previously [7]. Thermal cycling conditions were: reverse transcription at 45 °C for 10 min, initial denaturation at 94 °C for 10 min, followed by 45 cycles of [94 °C for 30 s, 60 °C for 60 s]. Positive and negative controls were included in each run. Rhinovirus-14, Strain 1059, ATCC, Manassas, VA, USA, propagated in HeLa Ohio-I cell line, was used as a positive control.

Amplification was performed using a real-time PCR detection system—Gentier 96R (Xi’an TianLong Science and Technology Co., Ltd., Shaanxi, China). Samples with a cycle threshold (ct) value < 38 were considered positive. From them, those with ct values < 25 were selected for further studies and genotyping. Samples where mixed infections with other respiratory viruses were detected were excluded from the study.

### 2.4. RT-PCR and Sanger Sequencing

Amplification of 5′UTR and VP4/VP2 region includes two separate RT-PCR assays. A seminested PCR protocol, targeting 5′UTR region was applied as described by Bochkov et al., with moderate modifications [8]. Reactions were performed using 3 µL extracted RNA, and forward and reverse primers at final concentration of 0.6 µM each in a total volume of 15 µL. Reverse transcription and first-round nested/seminested PCRs were performed simultaneously using QIAGEN One-Step RT-PCR Kit (Qiagen, Hilden, Germany). The reaction conditions were 50 °C for 30 min for reverse transcription, 95 °C for 15 min for reverse transcriptase deactivation, followed by 40 amplification cycles with denaturation for 30 s at 94 °C, annealing for 30 s at 50 °C, elongation for 90 s at 72 °C and a final extension at 72 °C for 10 min.

A mixture of six different forward primers (final concentration of 0.6 µM each), the same reverse primer and 5 µL of first-round PCR product were used in the second round of seminested PCR reaction. The amplification was performed using QIAGEN One-Step RT-PCR Kit (Qiagen, Hilden, Germany). The PCR reaction conditions were 94 °C for 3 min for initial denaturation, followed by 40 amplification cycles with denaturation for 30 s at 94 °C, annealing for 30 s at 52 °C, elongation for 60 s at 72 °C and a final extension at 72 °C for 10 min.

The protocol used allowed for the amplification of a DNA fragment of 370 bp. Sequencing of 5′UTR was performed using only reverse primer 5′UTR-revseq (sequence (5′ → 3′) TCAGGGGCCGGAGGA), described previously [8].

The amplification of the VP4/VP2 region followed a nested RT-PCR strategy. Reverse transcription and the first round of nested PCR were performed with the same reagents and reaction conditions as described above. Primers targeting VP4/VP2 region were F-458 and R-1125 [9,10].

Five microliters of first-round PCR product were used as a matrix in the second round of nested PCR reactions with primers F-547 and R-1087 [9,10]. The same primers were used for sequencing. The PCR reaction conditions were as described above except for the annealing temperature, which was set at 55 °C for 30 s. Nested PCR yielded a 540-bp amplification product.

All the PCR products were analyzed by agarose gel-electrophoresis and were sent for purification and sequencing by the Sanger method at Bioneer, Republic of Korea.

### 2.5. Genotyping and Sequence Analysis

All sequences were subjected to a BLAST search against RV reference sequences in the NCBI Nucleotide GenBank database for preliminary genotyping [11].

Genotype assignment for each sequence was based on: (1) best BLAST result hit to the whole genome sequence of reference strains, (2) analyzing the partial length of the VP4/VP2 sequences with web-based open-access enterovirus genotyping tool, version 1.0 [12] and (3) on the phylogenetic analysis of the sequences obtained in this study and sequences of respective regions of reference strains (downloaded from Genbank).

The complete RV genome sequences were retrieved from GenBank as references and aligned with the sequences obtained in this study via muscle algorithm for multiple sequence alignment. This alignment was again trimmed to set the sequence size determined in this study (approximately 200 bases for 5′UTR and 450 bases for VP4/VP2 region). The phylogenetic trees based on the 5′UTR and the VP4/VP2 region, respectively, were constructed using the Mega X software [13].

The 5′UTR tree was composed of forty-seven nucleotide sequences from this study and another eighty-nine RV isolates, with complete genome sequences available from databases. The phylogenetic tree was inferred using the maximum likelihood method and the general time reversible model [14] with 1000 bootstrap iterations.

The 5′UTR tree for RV-C was composed of 23 nucleotide sequences from this study and another 36 RV-C isolates with complete genome sequences, available on the GenBank nucleotide database. The evolutionary history was inferred using the neighbor-joining method [15] with 500 bootstrap replicates [16]. The evolutionary distances were computed using the Kimura 2-parameter method [14].

VP4/VP2 tree was composed of 38 nucleotide sequences from this study and another ninety RV isolates, with complete genome sequences available on the GenBank database. Phylogenetic tree was inferred using the maximum likelihood method, general time reversible model [17], with 1000 bootstrap iterations.

## 3. Results

### 3.1. RV Detections and Clinical Characteristics

#### 3.1.1. RV Detections

From January 2018 to the end of December 2019 and from January 2021 to the end of December 2021, 1385 nasopharyngeal swabs from patients were collected and tested for RVs by real-time RT-PCR. Of them, a total of 166 were positive. The overall positivity rate was 11.99% (166/1385). The number of RV detections varies over the three years included in this study as follows: 2018 (n = 361)—14.3% (51/361); 2019 (n = 517)—6.96% (36/517); 2021 (n = 507)—15.58% (79/507). A total of 63 samples out of 166 RV-positive clinical isolates were selected for further analysis and genotyping. Only 47 of them were successfully amplified, yielding sequences with quality good enough for phylogenetic analysis.

#### 3.1.2. Clinical Data

Descriptive clinical observations and diagnoses were obtained from the medical professionals sending the samples. For the purposes of the present study, recorded symptoms and diagnoses of successfully sequenced samples (n = 47), were coded as influenza-like infections—(i) ILI (acute onset within the last 10 days, fever ≥ 38 °C and cough), (ii) ARI (sudden onset and at least one of these: cough, sore throat, shortness of breath, coryza) and (iii) other. In order to investigate different RV types with consideration given to relative disease severity, and if there was information about it, cases of ARI were further subdivided according to upper respiratory tract (URTI) and lower respiratory tract (LRTI) involvement to ARI/URTI/Laryngitis, ARI/LRTI/Bronchiolitis, and ARI/LRTI/Croup. After the COVID-19 pandemic, detailed information on clinical manifestations and diagnosis were not provided and all respiratory samples received for testing at the NRL for Influenza and ARI were described as ILI/ARI-possible COVID-19. The samples were received with one of the following diagnoses (the number of samples with each diagnosis is indicated in parenthesis):

- ARI (n = 5);
- Acute respiratory distress (ARD) (n = 1);
- ARI/URTI/Laryngitis (n = 1);
- ARI/LRTI/Bronchiolitis (n = 13);
- ARI/LRTI/Croup (n = 2);
- ILI (n = 4);
- ILI/ARI-possible COVID-19 (n = 18);
- Other (n = 3).

#### 3.1.3. Diagnosis and Virus Type

The majority of patients were infected either with rhinovirus type A or type C. Significantly, higher prevalence of RV-A was found among patients with ILI (*p* = 0.0199), and RV-A (*p* = 0.0349) and RV-C (*p* = 0.0392) among ILI/ARI-possible COVID-19, when compared with patients infected with other rhinovirus types (Table 1).

#### 3.1.4. Virus Types before and after the COVID-19 Pandemic

When comparing the years before and after the COVID-19 pandemic, it became evident that RV-C detections were more common after the pandemic (*p* = 0.0189), while RV-A was detected more frequently before the pandemic (*p* = 0.0168). There were no significant differences in the detection of RV-B before and after the pandemic (Table 2).

#### 3.1.5. Phylogenetic Analysis and Genotype Assignments of RV Strains

**Figure 1:**
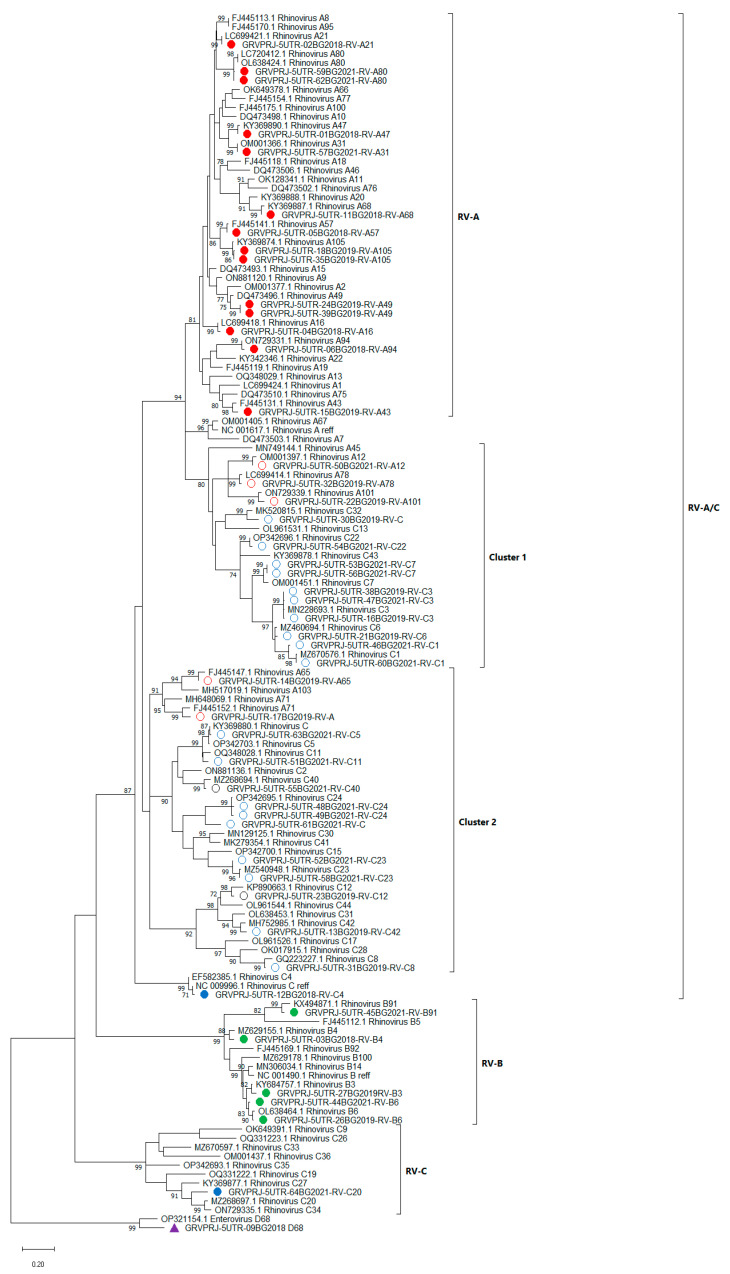
Phylogenetic tree based on the 5′UTR region of RV, constructed by the maximum-likelihood method with 1000 bootstrap iterations. Tree branches are proportional to genetic distance and all bootstrap values greater than 70 are shown at the branches. Reference sequences are represented by GenBank accession numbers and strains. Isolates from clinical samples are indicated by a sample ID number, the collection year and genotype. Strains most similar to RV-A reference strains are shown in red, strains most similar to RV-B reference strains are shown in green and strains most similar to RV-C reference strains are shown in blue. The strains in clusters 1 and 2 are indicated by circles. Enterovirus D68 was used as an outgroup. Enterovirus D68, identified in this study, is shown in purple.

All of the 47 RV isolates identified in this study were first classified based on the sequence of the 5′UTR. RV strains were classified into three species—RV-A, B and C, with the help of the enterovirus automated genotyping tool. Thus, 19 types were detected as RV-A, 5 as RV-B, and 23 as RV-C. However, this instrument was insufficient for further genotyping of the isolated viruses. For further detailed genotype assignments based on 5′UTR, BLAST score and phylogenetic analysis were combined and a divergence threshold of <7% was used [8]. All isolates were grouped into three genetic clades—RV-A/C, RV-B and RV-C. The first clade comprises RV strains similar to RV-A and RV-C with three distinct subgroups—RV-A types and two mixed clusters—cluster 1 and cluster 2 (Figure 1). 

Two strains (17BG2019 and 30BG2019) showed a close percentage of identity in BLAST analysis to both RV-A and RV-C, and therefore, could not be completely classified based on BLAST score only. Phylogenetic analysis grouped these isolates along with RV-A isolates in cluster 2 and along with RV-C isolates in cluster 1, respectively. However, these isolates showed divergence above 7% with their closest referent strains (Supplementary Table S1). Based on the BLAST score result, isolate 61BG2021 was classified as RV-C, and phylogenetic analysis grouped this isolate along with RV-C isolates in Cluster 2, but with divergence above 7% with the closest referent strains. These three isolates failed to be unequivocally genotyped. 

**Figure 2:**
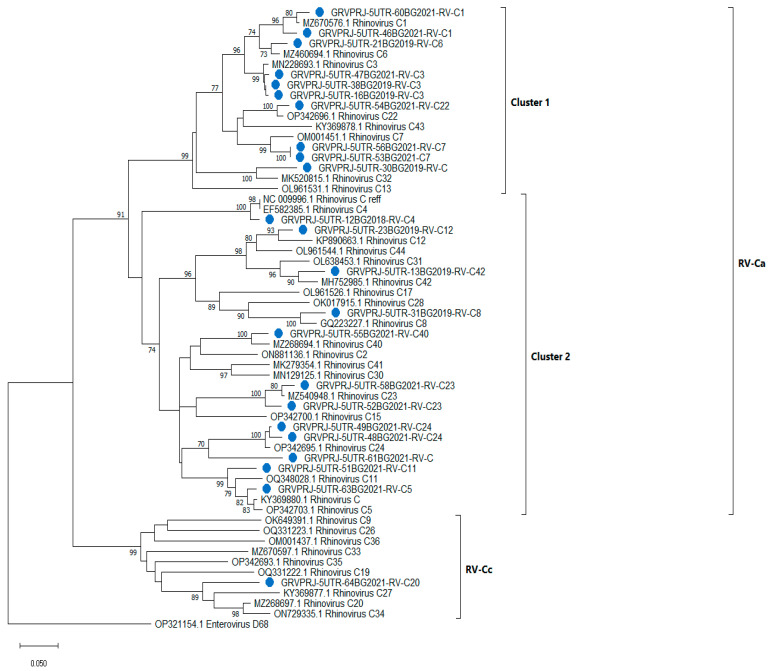
Phylogenetic tree based on the 5′UTR region of RV-C, constructed by the neighbor-joining method with 500 bootstrap iterations. Bootstrap values greater than 70 are shown at the branches. Reference sequences are represented by GenBank accession numbers and strains. Isolates from clinical samples are shown in blue and indicated by a sample ID number, the collection year and strains. Enterovirus D68 was used as an outgroup.

To investigate the relationship within the RV-C group, a neighbor-joining tree was constructed, including only RV-C sequences (n = 60). RV-C isolates were grouped into two subgroups—rhinovirus A-like (RV-Ca), comprising mixed clusters 1 and 2 and RV-Cc (Figure 2). Additionally, 61BG2021 did not group along any of the reference sequences, although it showed close relationships with RV-C24.

**Figure 3:**
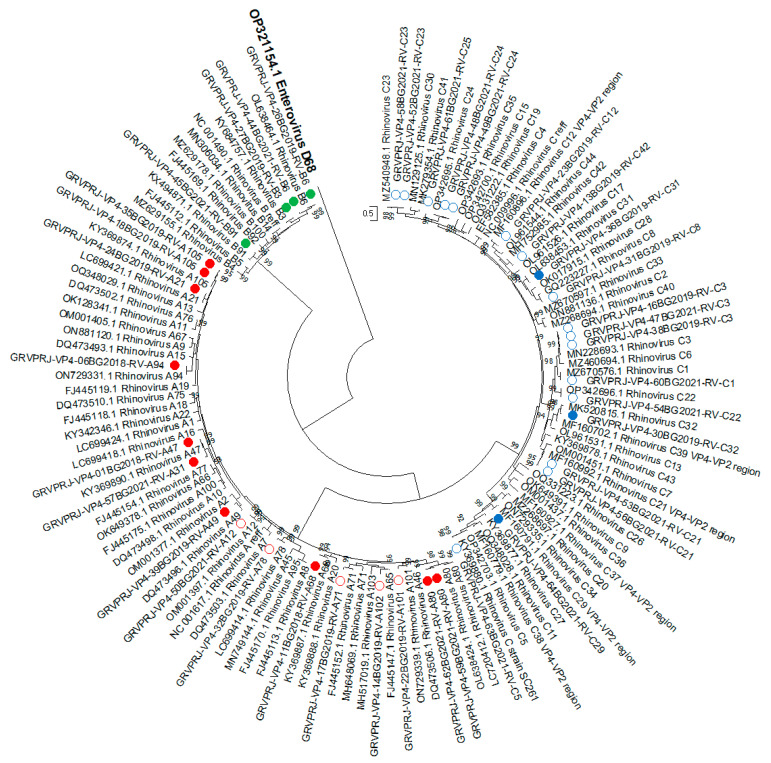
Phylogenetic tree based on the VP4/VP2 region of RV, constructed by the maximum-likelihood method with 1000 bootstrap iterations. Tree branches are proportional to genetic distance, and all bootstrap values greater than 90 are shown at the branches. Reference sequences are represented by GenBank accession numbers and strains. Isolates from clinical samples are indicated by a sample ID number, collection year and strain. Strains most similar to RV-A reference strains are shown in red, strains most similar to RV-B reference strains are shown in green, and strains most similar to RV-C reference strains are shown in blue. The strains in mixed clusters 1 and 2 from the previous analysis are indicated by circles. Enterovirus D68 was used as an outgroup.

All isolates were subjected to nested RT-PCR for amplification of the VP4-VP2 region, and 38 of them yielded amplicons, which were sequenced for further genotyping (GenBank ID: OQ849168–OQ849205). A Maximum likelihood tree was constructed based on the sequence of the VP4/VP2 region (Figure 3). Genotypes were assigned based on BLAST score results and phylogenetic grouping with a divergence threshold of <10% with the closest referent strain [4]. The result showed that fifteen of the isolates were RV-A, four were RV-B and nineteen were RV-C (Supplementary Table S1).

#### 3.1.6. Recombination Analysis

An analysis of the occurrence of recombination events between the 5′UTR and VP4/VP2 regions of the RV isolates was performed using 37 isolates, which yielded sequences of both regions. For three isolates, genotyping using 5′UTR showed different results compared to genotyping based on VP4/VP2 region. 24BG2019 was typed as RV-A, based on 5′UTR was grouped together with RV-A49, but based on VP4/VP2—with RV-A21. The 53BG2021 and 56BG2021 were genotyped as RV-C7 based on 5′UTR but as RV-C21 according to their VP4/VP2 sequences.

## 4. Discussion

The present study is the first to determine the different genotypes of RVs circulating in Bulgaria. Moreover, for the first time, the circulation of RV-C was demonstrated in the country. 

Respiratory disease surveillance established in Bulgaria does not routinely include testing for RVs. Nevertheless, until the COVID-19 pandemic, all samples from children under 5 years of age received in the NRL for Influenza and ARI were tested for influenza and other respiratory viruses, including RVs. This routine allowed for calculating the overall positivity rate of RV before the pandemic as 9.9% (for 2018 and 2019, combined) and after the pandemic as 15.58%. The post-pandemic period included testing all SARS-CoV2-negative samples for other respiratory viruses. As the age profile of patients who were tested changed over time, the age distribution of RV detections could not be compared between periods.

RV infections are more commonly associated with milder, cold-like symptoms. Such patients usually do not seek medical help. A limitation of this study is that RVs in our case were sought mostly among symptomatic patients with moderate or severe manifestations of their respiratory infection, for whom samples were forwarded to the reference laboratory. Therefore, the obtained results for RV genotypes probably do not entirely reflect the actual circulation pattern of RVs in Bulgaria.

In the results presented here, detections of RV-A were significantly more frequent in the years before the COVID-19 pandemic compared to the post-pandemic period, when RV-C prevailed. The interpretation of this observation should be conducted with particular caution, considering both the different ages of the patients and the differences in the clinical diagnoses.

It is known that RVs do not only cause common cold and influenza-like respiratory illnesses, but they are also associated with some lower respiratory tract diseases [3]. Our data also confirm the growing evidence that RV-C is associated with more severe respiratory disease with lower respiratory tract involvement [18,19,20,21]. Therefore, information about the RV genotype could be helpful for predicting the clinical severity of the infection.

RVs are known for their high genetic diversity. To date, over 165 RV genotypes have been described. RVs are grouped into three species designated as *Rhinovirus A* (RV-A)*, Rhinovirus B* (RV-B) and *Rhinovirus C* (RV-C). Previous studies have suggested that RV-C could be further classified into two subgroups: RV-Cc and RV-Ca, with the latter resulting from previous recombination with sequences related to RV-A [22,23]. Currently, type assignment is based on the pairwise identity of the nucleotide sequences of complete genomes, complete capsid proteins or VP1 genes [24]. Some studies also describe the use of the highly conserved nucleotide sequences of the 5′UTR and VP4/VP2 regions for rhinovirus subtyping [4,8,25,26].

In this study, two different approaches for amplifying and sequencing the 5′UTR and VP4/VP2 were combined, and results obtained from both regions were compared [8,9,10]. Although the VP1 region is largely accepted as suitable for typing all enteroviruses, including rhinoviruses, especially for RV-C, most sequence data available in GenBank have been collected in the VP4/VP2 region. According to Simmonds P. et al. [4], the classification of RV-C variants showing >10% divergence in VP4/VP2, but lacking VP1 sequences has to be in provisionally assigned types (subject to confirmation once VP1 sequences are determined). However, in our study, we did not identify any sequence with more than 10% divergence in the VP4/VP2 region; therefore, we assumed that genotype assignment is reliable.

Forty-seven of the RV positive samples were distributed into 34 genotypes from all the three RV species (See Supplementary Table S1). Phylogenetic analysis of the 5′UTR region grouped the isolates most similar to RV-A or RV-C in the BLAST search into mixed clusters within the RV-Ca subgroup. Sequences of the 5′UTR region alone were insufficient for correct genotyping of three of the isolates. This may be due to the evolutionary characteristics of the 5′UTR region, as well as the lack of complete genome sequences for some of the genotypes in the GenBank to be used as a reference. However, 10 out of 47 isolates were genotyped only based on their 5′UTR region, because nested RT-PCR failed to amplify their VP4/VP2 region. This also included one isolate that was typed as enterovirus D68. Interestingly, for this isolate real-time RT PCR was positive for RV. 

Thirty-eight isolates were completely distributed into RV-A, RV-B and RV-C in the phylogenetic tree based on the VP4/VP2 region.

Recombination plays a significant role in the evolution of RVs [27]. Comparison of phylogenetic grouping based on the 5′UTR and VP4/VP2 regions showed a different grouping for four isolates identified in this study. One was determined as RV-A49/A21, two as RV-C7/C21 and one as RV-C20/C29, respectively. Intratype recombination events may potentially be behind the difference in genotyping.

Samples from the present study were collected during the flu season, which we consider as a limitation. This did not allow for the estimation of RV circulation seasonality. 

Our observations indicate that multiple genotypes could be detected in Bulgaria from 2018 to 2021. The majority of circulating RV species were RV-A and RV-C. It was noticed that RV-A prevailed in the milder cases and RV-C in the more severe ones. Despite the limitations, the present study provides improved knowledge of the diversity and genetic characteristics of RVs.

## Figures and Tables

**Table 1:** Comparison of diagnoses related to virus type.

Diagnosis	RV-A n = 19	*p* ^1^	RV-B n = 5	*p* ^1^	RV-C n = 24	*p* ^1^
ARI	2 (10.5%)	n.s.	1 (20%)	n.s.	2 (8.3%)	n.s.
ARD	0	-	1 (20%)	n.s.	0	
ARI/URI/Laryngitis	0	-	1 (20%)	n.s.	0	
ARI/LRTI/Bronchiolitis *	6 (31.6%)	n.s.	0	-	6 (25%)	n.s.
ARI/LRTI/Croup	0	-	0	-	2 (8.3%)	n.s.
ILI	4 (21.1%)	0.0199	0	-	0	-
ILI/ARI-possible COVID-19	4 (21.1%)	0.0349	2 (40%)	n.s.	14 (58.3%)	0.0392
Other	3 (15.8%)	n.s.	0	-	0	-

^1^ Fisher’s exact test value. * One bronchiolitis sample was genotyped as enterovirus D68 and is not presented in the table. Abbreviations: n.s., not significant.

**Table 2:** Comparison of RV type detections before and after COVID-19.

Detection Period	RV-A n = 19	RV-B n = 5	RV-C n = 24
Before COVID-19	15	3	9
After COVID-19	4	2	15
Fisher’s exact test value	*p* = 0.0168	n.s.	*p* = 0.0189

Abbreviations: n.s., not significant.

## Data Availability

*Rhinovirus A*, *B* and *C* VP4/VP2 sequences, reported in this study were submitted to the GenBank sequence database under accession numbers OQ849168–OQ849205.

## Supplementary Materials

The following supporting information can be downloaded at: https://www.mdpi.com/article/10.3390/v15071608/s1, Table S1: Rhinovirus genotypes.

## References

[1] Makela M.J., Puhakka T., Ruuskanen O., Leinonen M., Saikku P., Kimpimaki M., Blomqvist S., Hyypia T., Arstila P. (1998). Viruses and Bacteria in the Etiology of the Common Cold. J. Clin. Microbiol..

[2] Fendrick A.M., Monto A.S., Nightengale B., Sarnes M. (2003). The Economic Burden of Non–Influenza-Related Viral Respiratory Tract Infection in the United States. Arch. Intern. Med..

[3] Hayden F.G. (2004). Rhinovirus and the Lower Respiratory Tract. Rev. Med. Virol..

[4] McIntyre C.L., Knowles N.J., Simmonds P. (2013). Proposals for the Classification of Human Rhinovirus Species A, B and C into Genotypically Assigned Types. J. Gen. Virol..

[5] Simmonds P., McIntyre C., Savolainen-Kopra C., Tapparel C., Mackay I.M., Hovi T. (2010). Proposals for the Classification of Human Rhinovirus Species C into Genotypically Assigned Types. J. Gen. Virol..

[6] Kiang D., Kalra I., Yagi S., Louie J.K., Boushey H., Boothby J., Schnurr D.P. (2008). Assay for 5′ Noncoding Region Analysis of All Human Rhinovirus Prototype Strains. J. Clin. Microbiol..

[7] Lu X., Holloway B., Dare R.K., Kuypers J., Yagi S., Williams J.V., Hall C.B., Erdman D.D. (2008). Real-Time Reverse Transcription-PCR Assay for Comprehensive Detection of Human Rhinoviruses. J. Clin. Microbiol..

[8] Bochkov Y.A., Grindle K., Vang F., Evans M.D., Gern J.E. (2014). Improved Molecular Typing Assay for Rhinovirus Species A, B, and C. J. Clin. Microbiol..

[9] Wisdom A., Leitch E.C.M., Gaunt E., Harvala H., Simmonds P. (2009). Screening Respiratory Samples for Detection of Human Rhinoviruses (HRVs) and Enteroviruses: Comprehensive VP4-VP2 Typing Reveals High Incidence and Genetic Diversity of HRV Species C. J. Clin. Microbiol..

[10] Lee W.-M., Kiesner C., Pappas T., Lee I., Grindle K., Jartti T., Jakiela B., Lemanske R.F., Shult P.A., Gern J.E. (2007). A Diverse Group of Previously Unrecognized Human Rhinoviruses Are Common Causes of Respiratory Illnesses in Infants. PLoS ONE.

[11] Camacho C., Coulouris G., Avagyan V., Ma N., Papadopoulos J., Bealer K., Madden T.L. (2009). BLAST+: Architecture and Applications. BMC Bioinform..

[12] Kroneman A., Vennema H., Deforche K., Avoort H.v.d., Peñaranda S., Oberste M.S., Vinjé J., Koopmans M. (2011). An Automated Genotyping Tool for Enteroviruses and Noroviruses. J. Clin. Virol. Off. Publ. Pan Am. Soc. Clin. Virol..

[13] Kumar S., Stecher G., Li M., Knyaz C., Tamura K. (2018). MEGA X: Molecular Evolutionary Genetics Analysis across Computing Platforms. Mol. Biol. Evol..

[14] Kimura M. (1980). A Simple Method for Estimating Evolutionary Rates of Base Substitutions through Comparative Studies of Nucleotide Sequences. J. Mol. Evol..

[15] Saitou N., Nei M. (1987). The Neighbor-Joining Method: A New Method for Reconstructing Phylogenetic Trees. Mol. Biol. Evol..

[16] Felsenstein J. (1985). Confidence Limits on Phylogenies: An Approach Using the Bootstrap. Evol. Int. J. Org. Evol..

[17] Nei M., Kumar S. (2000). Molecular Evolution and Phylogenetics.

[18] Miller E.K., Khuri-Bulos N., Williams J.V., Shehabi A.A., Faouri S., Al Jundi I., Chen Q., Heil L., Mohamed Y., Morin L.-L. (2009). Human Rhinovirus C Associated with Wheezing in Hospitalised Children in the Middle East. J. Clin. Virol..

[19] Bizzintino J., Lee W.-M., Laing I.A., Vang F., Pappas T., Zhang G., Martin A.C., Khoo S.-K., Cox D.W., Geelhoed G.C. (2011). Association between Human Rhinovirus C and Severity of Acute Asthma in Children. Eur. Respir. J..

[20] Lamson D., Renwick N., Kapoor V., Liu Z., Palacios G., Ju J., Dean A., St George K., Briese T., Lipkin W.I. (2006). MassTag Polymerase-Chain-Reaction Detection of Respiratory Pathogens, including a New Rhinovirus Genotype, That Caused Influenza-like Illness in New York State during 2004–2005. J. Infect. Dis..

[21] Linder J.E., Kraft D.C., Mohamed Y., Lu Z., Heil L., Tollefson S., Saville B.R., Wright P.F., Williams J.V., Miller E.K. (2013). Human Rhinovirus C: Age, Season, and Lower Respiratory Illness over the Past 3 Decades. J. Allergy Clin. Immunol..

[22] Ratnamohan V.M., Zeng F., Donovan L., MacIntyre C.R., Kok J., Dwyer D.E. (2016). Phylogenetic Analysis of Human Rhinoviruses Collected over Four Successive Years in Sydney, Australia. Influenza Other Respir. Viruses.

[23] McIntyre C.L., McWilliam Leitch E.C., Savolainen-Kopra C., Hovi T., Simmonds P. (2010). Analysis of Genetic Diversity and Sites of Recombination in Human Rhinovirus Species C. J. Virol..

[24] Harvala H., Broberg E., Benschop K., Berginc N., Ladhani S., Susi P., Christiansen C., McKenna J., Allen D., Makiello P. (2018). Recommendations for Enterovirus Diagnostics and Characterisation within and beyond Europe. J. Clin. Virol. Off. Publ. Pan Am. Soc. Clin. Virol..

[25] Adams M.J., Lefkowitz E.J., King A.M.Q., Bamford D.H., Breitbart M., Davison A.J., Ghabrial S.A., Gorbalenya A.E., Knowles N.J., Krell P. (2015). Ratification Vote on Taxonomic Proposals to the International Committee on Taxonomy of Viruses (2015). Arch. Virol..

[26] Wang W., He J., Liu Y., Xu L., Guan W., Hu Y. (2015). Molecular genotyping of human rhinovirus by using PCR and Sanger sequencing. Methods Mol. Biol..

[27] Palmenberg A.C., Spiro D., Kuzmickas R., Wang S., Djikeng A., Rathe J.A., Fraser-Liggett C.M., Liggett S.B. (2009). Sequencing and Analyses of All Known Human Rhinovirus Genomes Reveal Structure and Evolution. Science.

