# Supplementary material for: Rhinovirus Genotypes Circulating in Bulgaria, 2018–2021
Source: Viruses. 2023 Jul 22;15(7):1608. doi: 10.3390/v15071608 (PMC10385483; doi:10.3390/v15071608)
Supplement: Supplementary file 1 [file viruses-15-01608-s001.zip › viruses-2500188-supplementary.pdf]

Supplemetary Table S1. Rhinovirus genotypes

Isolate designation	Diagnosis	RV Type	Segment	Genotype	Div* <7%	Segment	Genotype	Div* <10%
**01BG2018**	ARI	RV A	5'UTR	A47	1.67%	partial	A47	1.15%
**02BG2018**	ILI	RV A	5'UTR	A21	1.26%	-	-	-
**03BG2018**	ARI	RV B	5'UTR	B4	1.69%	-	-	-
**04BG2018**	ILI	RV A	5'UTR	A16	2.49%	-	-	-
**05BG2018**	ILI	RV A	5'UTR	A57	2.08%	-	-	-
**06BG2018**	Other	RV A	5'UTR	A94	2.89%	VP4/VP2 partial	A94	0.76%
**11BG2018**	ARI/LRTI/Bronchiolitis	RV A	5'UTR	A68	2.05%	VP4/VP2 partial	A68	4.55%
**12BG2018**	ARI/LRTI/Bronchiolitis	RV C	5'UTR	C4	1.65%	-	-	-
**13BG2019**	ARI	RV C	5'UTR	C42	5%	VP4/VP2 partial	C42	6.92%
**14BG2019**	ARI/LRTI/Bronchiolitis	RV A	5'UTR	A102	0.82%	VP4/VP2 partial	A102	3.68%
**15BG2019**	ARI/LRTI/Bronchiolitis	RV A	5'UTR	A43	4.53%	-	-	-
**16BG2019**	ARI/LRTI/Bronchiolitis	RV C	5'UTR	C3	1.94%	VP4/VP2 partial	C3	3.43%
**17BG2019**	ILI	RV A	5'UTR	A71	8%↑	VP4/VP2 partial	A71	8.53%
**18BG2019**	Other	RV A	5'UTR	A105	2.48%	VP4/VP2 partial	A105	2.81%
**21BG2019**	ARI/LRTI/Bronchiolitis	RV C	5'UTR	C6	2.86%	-	-	-
**22BG2019**	ARI/LRTI/Bronchiolitis	RV A	5'UTR	A101	4.92%	VP4/VP2 partial	A101	8.97%
**23BG2019**	ARI/LRTI/Bronchiolitis	RV C	5'UTR	C12	5.74%	VP4/VP2 partial	C12	4.35%
**24BG2019**	ARI/LRTI/Bronchiolitis	RV A	5'UTR	A49	5.86%	VP4/VP2 partial	A21	1.77%
**26BG2019**	ARI/URI/Laringitis	RV B	5'UTR	B6	1.69%	VP4/VP2 partial	B6	0.69%
**27BG2019**	ARD	RV B	5'UTR	B3	2.44%	VP4/VP2 partial	B3	1.37%
**30BG2019**	ARI/LRTI/Croup	RV C	5'UTR	C32	8.92%↑	VP4/VP2 partial	C32	7.72%
**31BG2019**	ARI/LRTI/Bronchiolitis	RV C	5'UTR	C8	3.66%	VP4/VP2 partial	C8	6.55%
**32BG2019**	ARI/LRTI/Bronchiolitis	RV A	5'UTR	A78	3.27%	VP4/VP2 partial	A78	3.57%
**35BG2019**	Other	RV A	5'UTR	A105	2.44%	VP4/VP2 partial	A105	3%
**36BG2019**	ARI/LRTI/Bronchiolitis	RV C	-	-		VP4/VP2 partial	C31	0.7%
**38BG2019**	ARI/LRTI/Croup	RV C	5'UTR	C3	1.93%	VP4/VP2 partial	C3	2.89%
**39BG2019**	ARI	RV A	5'UTR	A49	2.09%	VP4/VP2 partial	A49	1.43%

**44BG2021**	ILI/ARI-possible COVID19	RV B	5'UTR	B6	3.81%	VP4/VP2 partial	B6	4.55%
**45BG2021**	ILI/ARI-possible COVID19	RV B	5'UTR	B91	4.17%	VP4/VP2 partial	B91	2.08%
**46BG2021**	ILI/ARI-possible COVID19	RV C	5'UTR	C1	5.53%	-	-	-
**47BG2021**	ILI/ARI-possible COVID19	RV C	5'UTR	C3	1.96%	VP4/VP2 partial	C3	4.43%
**48BG2021**	ILI/ARI-possible COVID19	RV C	5'UTR	C24	1.65%	VP4/VP2 partial	C24	2.91%
**49BG2021**	ILI/ARI-possible COVID19	RV C	5'UTR	C24	1.99%	VP4/VP2 partial	C24	1.86%
**50BG2021**	ILI/ARI-possible COVID19	RV A	5'UTR	A12	2.94%	VP4/VP2 partial	A12	2.85%
**51BG2021**	ILI/ARI-possible COVID19	RV C	5'UTR	C11	6.53%	-	-	-
**52BG2021**	ILI/ARI-possible COVID19	RV C	5'UTR	C23	5.39%	VP4/VP2 partial	C23	4.68%
**53BG2021**	ILI/ARI-possible COVID19	RV C	5'UTR	C7	4.49%	VP4/VP2 partial	C21	0.98%
**54BG2021**	ILI/ARI-possible COVID19	RV C	5'UTR	C22	1.99%	VP4/VP2 partial	C22	2.88%
**55BG2021**	ILI/ARI-possible COVID19	RV C	5'UTR	C40	3.7%	-	-	-
**56BG2021**	ILI/ARI-possible COVID19	RV C	5'UTR	C7	5.41%	VP4/VP2 partial	C21	0.98%
**57BG2021**	ILI/ARI-possible COVID19	RV A	5'UTR	A31	1.25%	VP4/VP2 partial	A31	0.71%
**58BG2021**	ILI/ARI-possible COVID19	RV C	5'UTR	C23	1.68%	VP4/VP2 partial	C23	3.6%
**59BG2021**	ILI/ARI-possible COVID19	RV A	5'UTR	A80	2.9%	VP4/VP2 partial	A80	7.72%
**60BG2021**	ILI/ARI-possible COVID19	RV C	5'UTR	C1	2.35%	VP4/VP2 partial	C1	0.36%
**61BG2021**	ILI/ARI-possible COVID19	RV C	5'UTR	C	-	VP4/VP2 partial	C25	6.99%
**62BG2021**	ILI/ARI-possible COVID19	RV A	5'UTR	A80	2.5%	VP4/VP2 partial	A80	7.02%
**63BG2021**	ILI/ARI-possible COVID19	RV C	5'UTR	C5	2.07%	VP4/VP2 partial	C5	6.09%
**64BG2021**	ARI	RV C	5'UTR	C20	6.25%	VP4/VP2 partial	C29	2.44%

* Divergence from the closest referent strain with complete genome, based on BLAST score result; Isolates for which 5′UTR region alone is insufficient for genotyping are marked in yellow; Isolates for which comparison of phylogenetic grouping based on the 5′UTR and VP4/VP2 regions showed a different grouping are marked in orange.
